# Insulinoma in childhood: a retrospective review of 22 patients from one referral centre

**DOI:** 10.3389/fendo.2023.1127173

**Published:** 2023-04-19

**Authors:** Maria Melikyan, Diliara Gubaeva, Anna Shadrina, Anna Bolmasova, Maria Kareva, Anatoly Tiulpakov, Artem Efremenkov, Yuri Sokolov, Klaus Brusgaard, Henrik T. Christesen, Kirstine Andersen, Alexey Stepanov, Julia Averyanova, Sergey Makarov, Larisa Gurevich

**Affiliations:** ^1^Department of Pediatric Endocrinology, Endocrinology Research Center, Moscow, Russia; ^2^Department of Endocrinology, Yerevan State Medical University, Yerevan, Armenia; ^3^Department of pediatrics, Center of Medical Genetics and Primary Health Care, Yerevan, Armenia; ^4^Department of Pediatric Endocrinology, Alder Hey Children’s Hospital, Liverpool, United Kingdom; ^5^Department of Endocrinology, Federal State Budgetary Scientific Institution Research Centre for Medical Genetics (RCMG), Moscow, Russia; ^6^Department of Pediatric Surgery, Central Clinical Hospital, Moscow, Russia; ^7^Department of Pediatric Surgery, Endocrinology Research Center, Moscow, Russia; ^8^Department of Clinical Genetics, Odense University Hospital, Odense, Denmark; ^9^Odense Pancreas Center OPAC and Steno Diabetes Center Odense, Odense, Denmark; ^10^Department of Endocrinology, Hans Christian Andersen Children’s Hospital, Odense University Hospital, Odense, Denmark; ^11^Department of Clinical Research, Faculty of Health Sciences, University of Southern Denmark, Odense, Denmark; ^12^Department of Abdominal Surgery, Russian Children's Clinical Hospital, Moscow, Russia; ^13^Morphological Department of Oncology, State Budget Health Agency Moscow Region Moscow Regional Research Clinical Institute, Moscow, Russia

**Keywords:** insulinoma, hyperinsulinemic hypoglycemia, Multiple Endocrine Neoplasia type 1 (MEN1), pancreatic NETs, malignant insulinoma

## Abstract

**Background:**

Insulinomas are very rare in childhood with sparse knowledge on the clinical aspects and the presence of Multiple Endocrine Neoplasia type 1 (MEN1).

**Methods:**

We conducted a retrospective review of patients diagnosed with insulinoma between 1995 and 2021, presenting to one referral centre in Russia. Clinical, biochemical, genetic, imaging and histological data were collected. In addition, follow-up and family data were obtained.

**Results:**

A total of twenty-two children aged 5 to 16 years were identified. The median (range) gap between the first hypoglycaemia symptoms and diagnosis was 10 (1–46) months. Twelve children (55%) were misdiagnosed to have epilepsy and were treated with anticonvulsants before hypoglycemia was revealed. Contrast enhanced MRI and/or CT were accurate to localize the lesion in 82% (n=18). Five patients (23%) had multiple pancreatic lesions. All children underwent surgical treatment. The median (range) diameter of removed tumors was 1.5 (0.3-6) cm. Histopathological studies confirmed the presence of insulinoma in all cases. Immunohistochemical studies revealed G2 differentiation grade in 10 out of 17 cases. Two patients were diagnosed with metastatic insulinoma. One of them had metastases at the time of insulinoma diagnosis, while the other was diagnosed with liver metastases eight years after the surgery. Eight children (36%) were found to carry *MEN1* mutations, inherited n=5, *de novo* n=1, no data, n=2. Children with MEN1 had significantly higher number of pancreatic tumors compared to sporadic cases. All of them developed additional MEN1 symptoms during the following 2-13 years. In the five patients with inherited MEN1, seven family members had hitherto undiscovered MEN1 manifestations.

**Conclusions:**

In this large cohort of children with rare pediatric insulinomas, MEN1 syndrome and G2 tumors were frequent, as well as hitherto undiscovered MEN1 manifestations in family members. Our data emphasize the need of genetic testing in all children with insulinoma and their relatives, even in the absence of any other features, as well as the importance of a prolonged follow-up observation.

## Background

Insulinomas are the most common functioning neuroendocrine tumors of the pancreas (pNET), although rare with an incidence of only 1-4 per million per year (1). An incidence peak is in the fifth decade, and insulinomas occur slightly more frequent in women (60%) (1, 2). In the pediatric population, insulinomas are even more rare. Most of the reports in the literature describe single pediatric clinical cases (3–11). There are only few pediatric cohorts of 9-10 cases reported (12, 13).

Insulinomas are usually well-differentiated benign tumors in the pancreas, but malignancy may occur in 5-10% (1). The majority of insulinoma cases are sporadic with only 5-10% of insulinomas being linked with genetic syndromes, of which Multiple Endocrine Neoplasia type 1 (MEN1) is the most common (14). Very few reports in the literature describe the association of insulinoma with neurofibromatosis 1 or tuberous sclerosis (14–16).

Clinically, insulinoma is characterized by recurrent episodes of hypoglycemia. Symptoms typically present after fasting or exercise, but may also develop postprandially (17–19). Biochemical diagnosis corresponds to the criteria of hyperinsulinemic hypoglycemia (HH) and can be established by the presence of detectable serum insulin and C-peptide levels (≥2 U/l and ≥ 0.6 ng/mL, respectively) taken during a hypoglycemic episode with glucose < 3.0 mmol/L. Recurrent hypoketotic hypoglycemia may lead to brain injury, especially in younger age.

In this single-center study, we describe an exceptionally large group of pediatric patients with insulinoma over a 26-year period.

## Materials and methods

### Study design

A retrospective review of the medical records of pediatric patients (age 0-18 years) diagnosed with insulinoma was performed. Insulinoma was diagnosed biochemically (serum insulin >2.0 U/l during the hypoglycemia <3.0 mmol/l) and by imaging (US, CT, MRI, endoscopic US), and verified by histopathology.

Collected data for the analysis included family history (parents were interviewed on known malignancies, benign lesions, ulcer, cholelithiasis or hypoglycemia in relatives), clinical symptoms prior to diagnosis, its onset and severity, results of the biochemical, hormonal, genetic and histopathological investigations. Whenever possible, clinical data during follow-up period were analyzed.

Screening for the signs of MEN1 syndrome included hormonal analysis (parathyroid hormone (PTH), cortisol, adrenocorticotropic hormone (ACTH), gastrin, prolactin, insulin growth factor 1 (IGF1)), imaging (brain MRI, abdomen US/CT, thyroid US), blood biochemical analysis (Ca, Ca++, glucose, ALT, AST), NET markers (serum chromogranin A and serotonin, urinal 5-Hydroxyindolacetic acid (5-HIAA)) and was performed at the first visit and during follow-up.

### Fasting test

Fasting test was performed according to the local protocol and required capillary glucose measurements using an automatic blood glucose meter for professional use every 3 hours if blood glucose (BG) was ≥4 mmol/l, every hour if BG was 3.5-3.9, and every 30 min. if BG was ≤3.4 mmol/l. A critical sample was obtained when BG was less than 3 mmol/L and included serum glucose, insulin and cortisol in all cases. Additionally, serum 3-hydroxybutyrate (BHB), and C-peptide, were measured in 15, and 13 cases, respectively.

### Biochemical and hormonal studies

Blood biochemistry was performed using Hitachi 912 Analyzer with standard reagents. Glucose was measured on plasma from venous blood samples with Cobas 8000 hexokinase assay analyzer (Roche^®^) with normal values 3.3 to 6.1 mmol/l. Bedside glucose values, or continuous glucose monitoring values, were not used for diagnostic fasting measurements. Serum BHB measurements were performed using a precision Xtra meter (Abbott Pharmaceuticals), with a reported assay range of 0 to more than 8 mmol/L. Urine ketone bodies were measured on an automated iChemVELOCITY analyser (Beckman Coulter Life Sciences, Krefeld, Germany) with urine test strips. Levels of insulin, C-peptide, cortisol, ACTH and PTH were measured using Cobas 6000 analyzer (Roche Diagnostic, Switzerland). Prolactin and IGF1 levels were measured using Vitros 3600 (Johnson & Johnson) and a Liason (DiaSorin) analyzer, respectively. Serum NET markers were evaluated using standard immunoassay method, urine 5-HIAA — using liquid chromatography method.

### Histological studies

Histological and immunohistochemical (IHC) studies were performed on sections 3–5 µm thick prepared from paraffin blocks. For IHC studies, an Autostainer (Autostainer, LabVision, type 480s, UK) was used. Sections were deparaffinized and antigenicity was restored in buffer pH 9.0 in a PT Module (Thermo Scientific, UK). To determine the type of tumor, a spectrum of antibodies was used: Chromogranin A (clone LK2H10), synaptophysin (clone MRQ-40), CD56 (clone MRG42), insulin (polyclone, RTU), glucagon (polyclone, RTU), somatostatin (polyclone, RTU, Cell Marque, USA), and gastrin (all from Cell Marque, USA), pancreatic polypeptide (clone EPR2330-10, Abcam, USA), Ki67 (clone MIB1, DAKO), somatostatin receptors type 2 (rabbit monoclonal EP149, Epitomix, USA) and type 5 (rabbit monoclonal UMB4, Epitomix, USA).

Somatostatin receptor (SSTR) expression analysis was performed according to the method of Volante M. et al.(20). Membrane expression of SSTR2, or membrane-cytoplasmic expression of SSTR5, was considered as positive if found in more than 30% of tumor cells.

The expression of other cytoplasmic markers was estimated according to standard semi-quantitative method for cytoplasmic markers. Tumor grade was assessed using Ki67-index according to the World Health Organization guideline (21).

### Genetic studies

Genomic DNA from peripheral blood leukocytes was extracted using standard methods (22). Molecular genetic analysis of *MEN1* was performed using bidirectional direct sequencing (n=21). Samples were sequenced in both directions using the BigDye Terminator v3.1 Cycle Sequencing Kit (Applied Biosystems, FosterCity, CA, USA) and analyzed on a ABI3730XL DNA Analyzer (AppliedBiosystems, Naerum, Denmark). Sequence analysis was performed using SeqMan Software (DNASTAR, Madison, WI, USA). To detect larger deletions Multiplex Ligation-dependent Probe Amplification (MLPA) analysis was performed (n=4) according to the manufacturer’s recommendations (Salsa MLPA, P017 MEN1, MRC-Holland, Amsterdam, the Netherlands). Data were analyzed using GeneMarker (Softgenetics, Pennsylvania, USA). *MEN1* DNA variant nomenclatures were given according to GenBank accession no. NM_000244.4.

### Follow-up studies

Follow-up investigations included screening for MEN1 components as described for the first visit, including detection of any distant metastases, using abdominal ultrasound or MRI. In patients with genetically verified MEN1 syndrome first grade relatives (parents and siblings) underwent genetic testing for point mutation in *MEN1* gene and in case of positive results, — screening for MEN1 components as mentioned above.

### Statistics

Demographic and clinical data were presented as median (interquartile range (IQR)). Statistical analysis was performed using StatSoft Inc., USA, version 10.0. Non-binary data were analyzed with the help of Mann-Whitney U test and chi-square test with p value < 0.05 considered as significant.

## Results

### Patient demographics and characteristics

We analyzed 465 medical records of children aged less than 18 years admitted to the Endocrine Research Center with HH from 1995 to 2021. A total of 22 patients (13 females) were diagnosed with primary pancreatic insulinoma, accounting for 4.7% of all pediatric patients with HH.

The median age at the time of first symptoms was 10.45 years. Median age at the time of diagnosis was 11.5 years, giving the median delay in diagnosis of 10 months (**Table 1**). Twelve children (55%) were misdiagnosed to have epilepsy and were treated with anticonvulsants before the hypoglycemia was revealed. All patients had typical clinical features of hypoglycemia, including drowsiness (73%), seizures (73%), syncope (68%), progressive weight gain (45%), learning and behavioral difficulties (45%). A third of the patients experienced hypoglycemic coma prior to diagnosis (7/22; 32%).

**Table 1:** Main clinical and biochemical parameters of 22 pediatric patients with insulinoma.

Characteristics	Results
Male : Female, absolute numbers	9:13
Age at the onset	10.45 (5.1-16.2) years
Age at the time of diagnosis	11.5 (7.7-16.8) years
Fasting test duration*	8 (1–19) hours
Serum glucose level at the end of the fasting test*	1.9 (0.5-2.2) mmol/L
Serum insulin level at the end of the fasting test*	20.9 (8.13-149) U/L
Serum C-peptide level at the end of the fasting test**	3.4 (1.96-10.2) ng/mL

All data are given as median (range) if not otherwise indicated. *Performed in 21/22 cases. **Performed in 13/22 cases.

### Hypoglycemia evaluation

Median duration of diagnostic fasting test was eight hours (**Table 1**). In all cases it resulted in laboratory hypoglycemia with a mean serum glucose level of 1.76 ± 0.63 mmol/L. Median serum insulin level taken during hypoglycemia was 20.9 U/L, median C-peptide level 3.4 ng/mL (n=13). Serum hydroxybutate was less than 0.5 mmol/l in all cases (n=15). Urine ketone bodies were undetectable in all measurements in cases when serum ketones were not available (n=7).

### Insulinoma imaging

**Figure 1:**
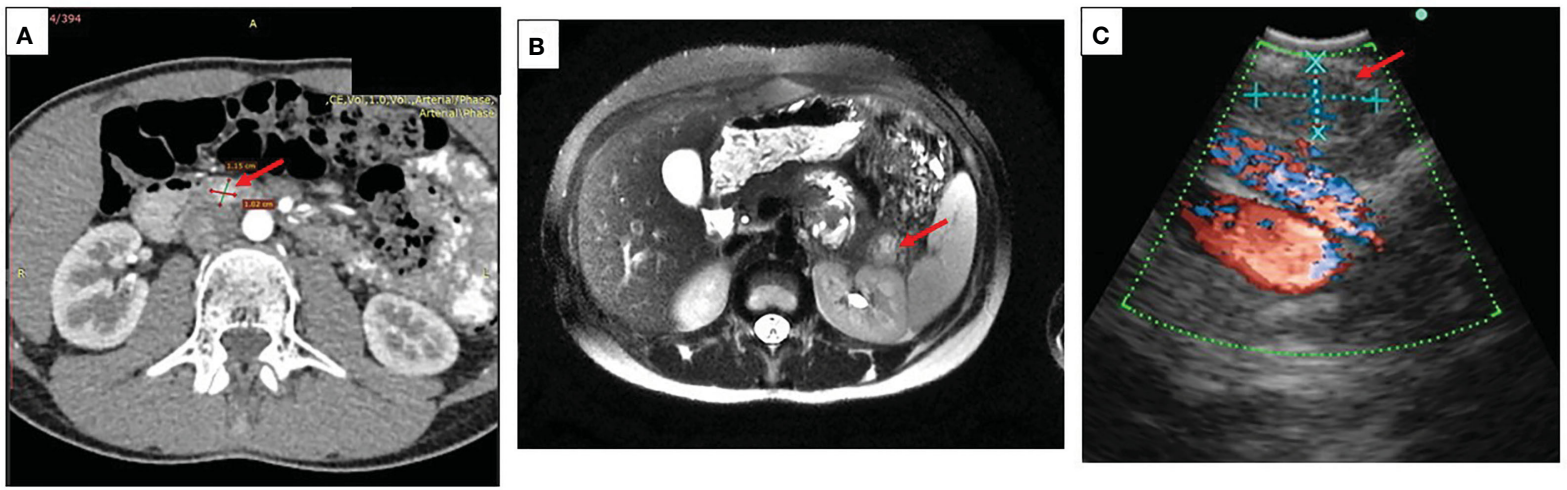
Imaging in pediatric patients with insulinoma **(A)** A representative CT scan showing insulinoma in the head of the pancreas (case #14), arrow indicating a round shaped hypervascular 1.5 cm lesion in the head of the pancreas highly enhanced during the arterial phase; **(B)** A representative contrast MRI scan showing an insulinoma in the pancreatic tail (case #8), arrow indicating a 2.3 cm lesion in the pancreatic tail with high signal intensity on T2 MR regimen; **(C)** A representative picture of endoscopic ultrasound localizing lesion in the pancreatic tail (case #13), arrow indicating a 0.9 cm ovoid shaped lesion, isoechoic with hypoechoic inclusions and distinct margins.

Localization of the tumor only by transabdominal ultrasound was possible in seven patients. Contrast enhanced CT and/or MRI were used in 18 cases. Additional endoscopic ultrasound was needed in four cases with inconclusive results of MRI and CT. **Figure 1** represents results of different imaging technics used for the evaluation of pancreatic lesions in our cohort of patients.

Five children (23%) had multiple pancreatic lesions. The 30 lesions were found equally in all parts of the pancreas (tail; n=11, head; n=10, body; n=9).

### Hypoglycemia management

Prior to surgery, 12 children required hyperglycemic medication. Diazoxide and octreotide alone were used in nine and two cases, respectively. One patient (case#12) had a combination of both drugs. Doses of diazoxide ranged from 100 to 300 mg/day. In 7 out of 10 patients, diazoxide was sufficient to maintain normoglycemia. Octreotide was given by subcutaneous injections every 8 hours in doses of 400 mcg/day. Other patients were managed with frequent feeds and/or continuous dextrose infusion.

### Pancreatic surgery

Surgical treatment was performed in all children: insulinoma enucleation in 11 (50%), partial pancreatic resection in 8 (36%), subtotal pancreatectomy in 2 (9%). In one patient (5%) with multiple lesions, repeated surgeries ended up with pancreatogastroduodenal resection (**Table 2**). Removed tumors varied in size from 0.3 to 6 cm. Six tumors were less than 1 cm, 14 ranged from 1 to 2 cm, and 10 were larger than 2 cm.

**Table 2:** Comprehensive clinical data on 22 pediatric patients with insulinoma and their family members.

Case	MEN1 mutation, Inheritance	Age at onset, years	Age at diagnosis, years	Treatment prior to surgery	Type of surgery	Amount of pNETs (n) and size	Grade	Follow- up duration, years	Follow-up findings and treatment	Family history	Family members’ investigation results
1	с.830C>G p.P277R Paternal	9.5	11.3	Frequent feeds	Partial resection	N=2. ⌀ 1.2cm; 0.3cm	G2	4.2	Pituitary adenoma at 13 years; hPTH at 15 years	Peptic ulcer in father	hPTH in father, hPRL in cousin
2	с.1547insC, p.(Lys517Glufs*14) Unknown inheritance	7.3	8.3	Glucose infusion	Subtotal pancreatectomy	N=1. ⌀ 2.5cm	No data	17.5	hPRL at 21 years (on Cabergoline); Gastrinoma at 25 years (surgical treatment).	Gastric cancer in grandmother	No data
3	с.936delC, p.(Tyr313Ilefs*55) Paternal	9.4	13.2	Frequent feeds	Enucleation	N=1. ⌀ 3cm	No data	6.1	hPTH, hPRL, adrenal nodular hyperplasia at 19 years	Unremarkable	hPTH in father and brother
4	c.784-9G>A p.? HGMD no. CS991446 Maternal	8.2	10.9	Frequent feeds	Enucleation	N=2. ⌀ 1.5cm; 1.4cm	G2	5.2	hPTH at 13 years; hPRL at 16 tears (on Cabergoline).	Unremarkable	Unremarkable
5	c.625_628delACAG p.T210Sfs*13 Paternal	5.1	8.8	DZX	Partial resection	N=3. ⌀ 2.0 cm; 0.6 cm; 0.5cm.	G1	5.3	Somatotropinoma at 12 years; pNET at 13 years; hPTH at 14 years. Underwent parathyroidectomy and second pancreatic surgery (previously on SST analogues for 3 years).	Unremarkable	Glucose intolerance, hypercalcemia in father
6	c.923C>A p.S308* HGDM no. CM970932 Unknown inheritance	13.3	13.8	DZX	Enucleation	N=2. ⌀3.7 cm; 0.6 cm	G2	4.5	hPTH at 18 years	Father died at 35 years (reason unknown)	No data
7	c.133G>A p.E45K Paternal	7.1	7.7	DZX	Partial resection	N=1. ⌀ 1.1cm	G2	1	None	Unremarkable	hPTH in father
8	c.141dup p.Leu48Serfs*69 DeNovo	11.4	11.5	Frequent feeds	Enucleation	N=1. ⌀ 2.3cm	G2	0.5	None	Unremarkable	No data
9	Negative	16.2	16.8	Frequent feeds	Enucleation	N=1. ⌀3.5cm	G2	1.5	None	Colon cancer in grandmother; Lung cancer in father`s sister at 25 years	No data
10	Negative	11.2	12.6	Glucose infusion	Enucleation	N=1. ⌀ 3.0cm	G1	2.2	None	Colorectal cancer in mother`s brother at 42 years	No data
11	Negative	10.8	11.5	Frequent feeds	Enucleation	N=1. ⌀ 1.5cm	G2	2.1	None	Unremarkable	No data
12	Not done	11	11.1	Octreotide + DZX + glucose infusion	Subtotal pancreatectomy + splenectomy	N=1 ⌀ 6cm + Multiple liver mts	G2 in tumor G3 in mts	0.5	Deceased at 11 years 8 months. Previously treated with SST + mTOR inhibitors.	Unremarkable	No data
13	Negative	7.3	7.7	DZX	Enucleation	N=1. ⌀ 0.95cm	G1	No data	No data	Unremarkable	No data
14	Negative	12.3	14.5	Octreotide	Pancreatic resection	N=1. ⌀ 1.56cm	G1	No data	No data	Peptic ulcer in mother; Hypoglycemia ()? in maternal cousin	No data
15	Negative	13.5	14.1	DZX	Partial resection	N=1. ⌀ 2cm	G1	1.3	Kreon	Pancreatic cancer in maternal grandmother	No data
16	Negative	12.4	13	Octreotide	Partial resection	N=1. ⌀ 1.5cm	G2	0.5	None	Gastric cancer in maternal sister	No data
17	Negative	16.1	16.3	DZX	Partial resection	N=1. ⌀ 1.1cm	G1	No data	No data	Gastric and colon cancer in paternal grandfather	No data
18	Negative	10.1	13.8	Frequent feeds	Enucleation	N=1. ⌀ 1.9cm	No data	5.1	Epilepsy at 18 tears	Unremarkable	No data
19	Negative	7.3	11	Frequent feeds	Enucleation	N=1. ⌀ 1cm	No data	4.5	None	Rectal cancer in grandfather	No data
20	Negative	10.1	11.2	DZX	Enucleation at 11 years; Partial resection at 12 years; Pancreato-gastro-duodenal resection at 13 years	N=4. ⌀ 1.5cm; 0.5cm; 1.0cm; 3.0cm.	G1-G2	21.5	Postoperative DM; Liver mts at 21 years; Nephropathy at 32 years. Treated with SST analogues, mTOR inhibitors, dialysis.		No data
21	Negative	8.0	9.1	DZX	Enucleation	N=1. ⌀ 1.9 cm	G1	No data	No data	No data	No data
22	Negative	15.8	16.5	DZX + glucose infusion	Partial resection	N=1. ⌀ 2.5 cm	No data	No data	No data	No data	No data

DZX, diazoxide; pNET, pancreatic neuroendocrine tumor; N, number of pNETs; ⌀, diameter; mts, metastasis; G1, low tumor grade; G2, intermediate tumor grade; SST, somatostatin; hPTH, hyperparathyroidism; hPRL, hyperprolactinemia; HGMD, Human Gene Mutation Database; ^®^, mTOR inhibitors, mammalian target of rapamycin inhibitors; DM,m diabetes mellitus.

### Histopathology results

**Figure 2:**
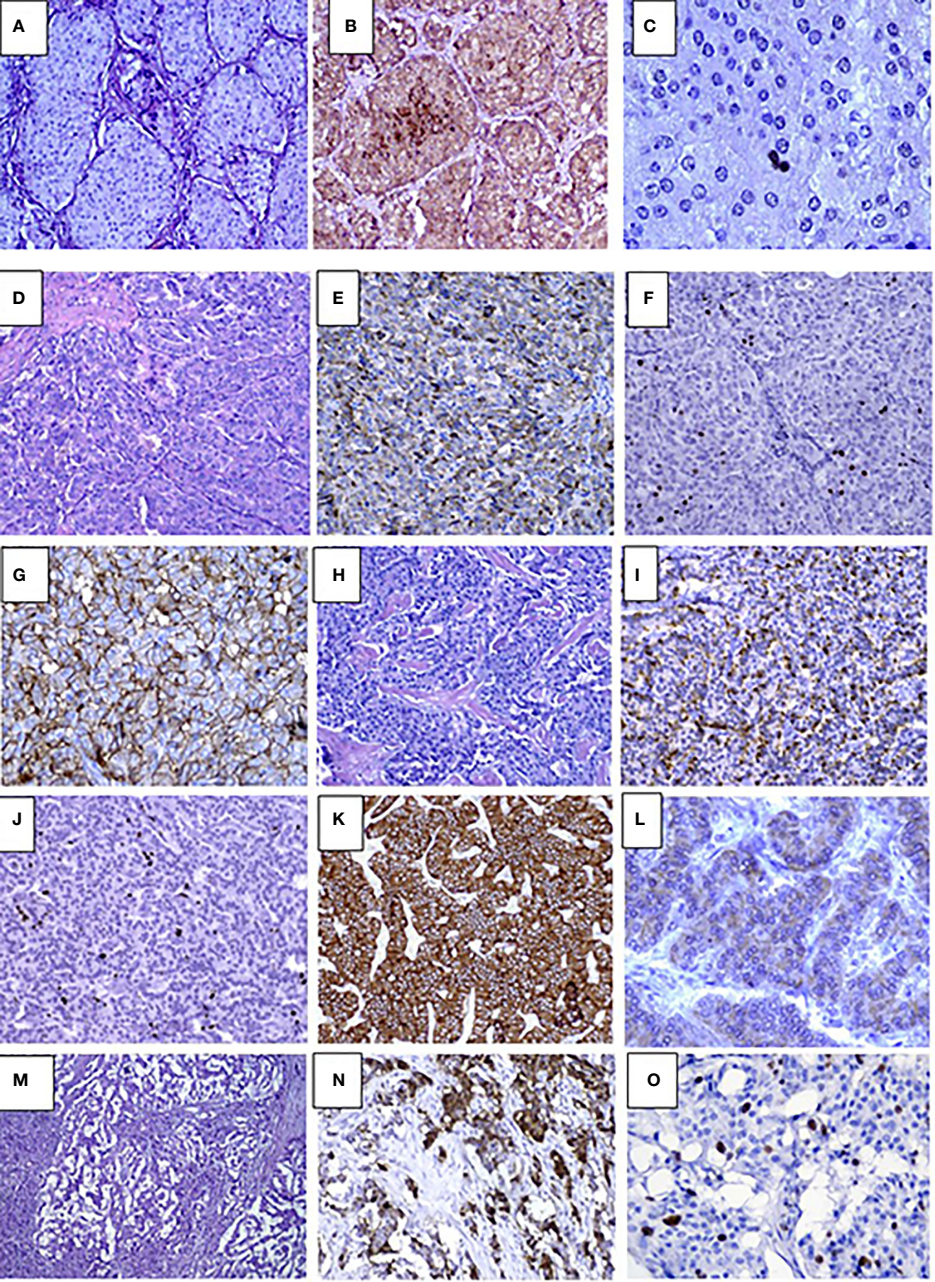
Pathology results in pediatric patients with insulinoma **(A–C)**. Case #10. Solid G1 insulinoma composed of packets of monomorphic cells, H&E, х250 **(A)**, Immunohistochemistry staining with insulin shows abundance of insulin secreting cells within the lesion, х250 **(B)**, Ki67 positive staining in single nuclei of tumor cells (1%) х400 **(C)**. D-L. Case #4. Trabecular G2 insulinoma with poorly developed stroma, H&E, х250 **(D)**, insulin staining, x250 **(E)**, Ki67 staining х250 **(F)**, membrane expression of SSTR type 2 in tumor cells (3+), x250 **(G)**; trabecular glucagon positive and insulin negative NET, G2, H&E, x250 **(H)**, moderate apical glucagon expression in tumor cells, glucagon staining x 250 **(I)**, Ki67 staining, x250 **(J)**, abundant membrane expression of SSTR type 2 (3+), x250 **(K)**, mild expression of SSTR type 5 (2+), x400 **(L)**, M-O. Case #12. Multifocal invasive insulinoma with trabecular architecture G2, H&E, x250 **(M)**, large complexes and small tumor cell nests expressing insulin, Insulin staining, x250 **(N)**, intensive Ki67 expression in cell nuclei, Ki67 staining, x400 **(O)**.

Histopathological studies confirmed the presence of insulinoma in all cases. Lesions showed polymorphic histology: most of the tumors had trabecular architecture (**Figures 2D–O**), rarely solid (**Figures 2A–C**) or mixed. Majority of lesions were encapsulated and well circumscribed from the surrounding pancreatic tissue. One patient (case #12) had multifocal insulinoma without capsule and invasion to the surrounding tissue (**Figures 2M–O**). This patient had liver metastases at the time of diagnosis.

Immunohistochemical studies were performed in 17 cases. All lesions demonstrated the expression of Synaptophysin and Chromogranin A. Among the children with multiple pancreatic lesions (n=5), some of the NETs were negative for insulin staining. Of these, glucagon expressing tumors were found in two cases (**Figures 2H–L**). Others were classified as non-functioning. Compound expression of gastrin and insulin was found in 1 case. SSTR2 and SSTR5 expression was analyzed in 13 lesions and was positively expressed in 6 and 5 cases, respectively (**Figures 2G, K, L**). Ki67 index and/or mitotic indexes were measured in a total of 19 lesions. In ten cases we found G2 differentiation grade (**Table 3**).

**Table 3:** Pathology results for 22 pediatric patients with resected insulinoma.

Case	n of lesions	Max Size in Diameter (cm)	localization	Morphology features	Immunohistochemistry							
					Syn	CgA	Ins	Gastr	Gluc	SSTR2/SSTR5	Ki67 (%)	Grade
1	2	1.2	Head	solid	+	+	+	-	-	ND	3.5	G2
		0.3	Tail	solid	+	+	+	-	-	ND	ND	ND
2	1	2.5	Head	ND	ND	ND	ND	ND	ND	ND	ND	ND
3	1	3	Tail	mixed	ND	ND	ND	ND	ND	ND	ND	ND
4	2	1.5	Body	solid	+	+	+	-	-	+/+	11	G2
		1.4	Tail	trabecular	+	+	-	-	+	+/+	12	G2
5	3	2	Body	mixed	+	+	+	-	-	-/+	1	G1
		0.6	Tail	solid	+	+	+	-	-	-/-	1	G1
		0.5	Head	solid	+	+	-	-	-	-/-	1	G1
6	2	3.7	Head	Solid	+	+	+	-	-	+/+	11	G2
		0.6	Tail	trabecular	+	+	-	-	+	-/-	8	G2
7	1	1.1	Tail	mixed	+	+	+	ND	ND	-/-	2	G2
8	1	2.3	Tail	trabecular	+	+	+	-	-	-/-	10.5	G2
9	1	3.5	Tail	mixed	+	+	+	-	-	+/-	10.5	G2
10	1	3.0	Tail	solid	+	+	+	-	-	ND	1	G1
11	1	1.5	Body	trabecular	+	+	+	+	-	+/-	4	G2
12	1	6	Body	trabecular	+	+	+	-	-	ND	8% in the tumor, 21.5% in mts	G2 in tumor, G3 in Mts
13	1	0.95	Tail	mixed	ND	ND	+	ND	ND	ND	1	G1
14	1	1.56	Head	mixed	+	+	+	-	-	ND	1	G1
15	1	2	Head	trabecular	+	+	+	-	-	ND	1	G1
16	1	1.5	Head	mixed	+	+	+	ND	ND	ND	4	G2
17	1	1.1	Tail	trabecular	+	+	+	-	-	+/+	1	G1
18	1	1.9	Head	ND	ND	ND	ND	ND	ND	ND	ND	ND
19	1	1	Body	solid	ND	ND	+	ND	ND	ND	ND	ND
20	4	1.5	Head	trabecular	ND	ND	+	ND	ND	ND	ND	G1
		0.5	Head	ND	ND	ND	ND	ND	ND	ND	ND	ND
		1	Body	ND	ND	ND	ND	ND	ND	ND	ND	ND
		3	Body	mixed	ND	ND	ND	ND	ND	ND	ND	G2
21	1	1.9	Body	trabecular	+	+	+	-	-	-/-	1	G1
22	1	2.5	Body	mixed	+	+	+	ND	ND	ND	ND	ND

ND, no data; SYN, synaptophysin; CgA, chromogranin A; Ins, insulin; Gastr, gastrin; Gluc, glucagon; SST2, somatostatin receptors type 2; SST5, somatostatin receptors type 5; mts - metastases. “+”, positive staining; “-”, - negative stainingm.

### MEN1 evaluation

We performed biochemical screening for the MEN1 syndrome components at the time of insulinoma diagnosis. This revealed mild normocalcemic hyperparathyroidism in two patients (cases #4 and #5), mildly elevated serotonin in three patients (cases #4,5,6), and high levels of Chromogranin A and 5-HIAA in one (case #6). There were no biochemical or radiological signs of pituitary adenoma. Analysis of the family history was possible in 20 cases and revealed malignancies in relatives (n=6), ulcer (n=2) and hypoglycemia (n=1). None of the interviewed relatives had a history of hyperparathyroidism or insulinoma (**Table 2**).

*MEN1* gene sequencing revealed pathogenic variants in 8 out of 21 children (38%). Of them, 2 missense, 1 nonsense, 1 splicing and 4 frameshift mutations were identified (**Table 2**). Five variants were previously described in MEN1 patients (23–27). Mutations p.P277R (case #1), p.Tyr313Ilefs*55 (case #3), and p.Leu48SerfsTer69 (case #8) are novel. With regards to a latter variant, a different substitution in the same codon was previously described in the literature as disease causing (28).

We compared main clinical characteristics in children with sporadic insulinomas (n=13) and those with pathogenic variants in the *MEN1* gene (n=8) (**Table 4**). Children with MEN1 syndrome tended to be younger at the time of insulinoma presentation, with a higher number of lesions and more often G2 grade. Significant difference between the two groups was, however, only found in the number of lesions (**Table 4**).

**Table 4:** Comparative analysis of the clinical features in pediatric patients with sporadic vs. MEN1-associated insulinoma.

	Sporadic insulinoma n=13	MEN1 syndrome n=8	p
Male : Female ratio	5:8	3:5	N.S.
Age at onset, years Median [Q25-Q75]	11.2 [10.1-13.5]	9.3 [7.2-9.45]	N.S.
Multiple pancreatic lesions (patient n, %)	1/13 (7.7%)	4/8 (50%)	p=0.028
Tumor size, cm Median [Q25-Q75]	1.53 [1.07-2.12]*	1.4 [0.6-2.3]**	N.S.
G2 grade n/n (%)	4/10 (40%)	5/6 (83.3%)	N.S.
Serum insulin (U/L) during hypoglycemia Median [Q25-Q75]	21.84 [14.4-41.1]	15.9 [12.1-30.4]	N.S.

N.S., Non significant. *n=16, **n=13.

Genetic testing of parents was performed in six families and revealed MEN1 carriers in five. All relatives with *MEN1* mutations were investigated. Despite the absence of suspicious family history and minimal clinical presentations, four of out the five *MEN1* parental carriers and two additional relatives were found to have components of MEN1 syndrome, but none compatible with insulinoma (**Table 2**).

Follow-up data were available in 17 patients. Median [25-75%] follow-up duration was 4.2 [1.3-5.2] years. There was no recurrence of insulinoma during follow-up. All children with genetically confirmed MEN1 syndrome developed various MEN1 components during next 2-13 years with hyperparathyroidism and hyperprolactinemia being the most common findings (n=5 and 3 resp.), (**Table 2**). In one patient, liver metastases were found eight years after the pancreatectomy (case #20).

## Discussion

While congenital hyperinsulinism is the most common cause for persistent and recurrent hypoglycemia in infancy (29), the possibility of insulinoma should be considered in those with HH presenting after the age of three years. In our group, the youngest age at onset was five years. Literature reports describe cases of insulinoma in even younger children (12). As well as others, we noticed a pronounced delay in diagnosis, which was approximately a year since the first symptoms of hypoglycemia (12). The delay can be explained by the extreme rarity of the condition and the nonspecific, episodic symptoms. Most of the children in our group experienced hypoglycemia only after prolonged fast. In contrast to adults, children with insulinoma tend to develop neuroglycopenic symptoms of hypoglycemia more often (3). In our cohort, 73% of patients had hypoglycemic seizures that led to a misdiagnosis of epilepsy in a half of cases.

According to a recent review, routine imaging techniques such as transabdominal ultrasound, CT and MRI have relatively low accuracy for insulinoma localization with estimated sensitivity of 9-66%, 35-82% and 35-63%, respectively (30). Endoscopic ultrasound seems to be the most accurate diagnostic tool for insulinomas with a sensitivity of 94% alone or up to 100% if combined with CT scan (31). Recently invented imaging technics such as 68Ga-DOTATATE PET and GLP-1 receptor scintigraphy are widely used in patients where the first-line imaging tests are unable to detect the lesion (32).

In our cohort, routine imaging technics were accurate in 86% of cases. This finding fits with previous publications on pediatric insulinomas where MRI alone localized pancreatic lesions in 88% (7/8) of patients (12). The higher imaging sensitivity compared to adults may be related to a bigger tumor size in the pediatric cohorts. For instance, in our group lesions were ≥1 cm in diameter in 80% and ≥ 2 cm in 33% of cases, whereas in adults insulinomas usually do not exceed 1 cm (33). We speculate that insulinomas in childhood grow more rapidly, rather than having a longer diagnostic delay, compared to insulinomas in adults.

There is an association between size of the tumor and its malignancy potential (34). Malignancy of the insulinoma is only defined by the presence of metastases or the invasion in surrounding organs (35). Malignant insulinoma is rare and accounts for only 5-10% of all cases of insulinoma (36) with only few reports of pediatric cases in the literature (4, 7, 37). Insulinomas are usually classified using the 2017 WHO grading system which is mainly based on mitotic and/or Ki67 index (21), although a new classification has recently been suggested (38). According to the latter insulinomas can be divided into two subtypes: “Typical” insulinomas that have strong epigenetic similarities to pancreatic beta-cells (*PDX1*-positive/*ARX*-negative) and a favorable prognosis after the complete surgical resection. These typical insulinomas become symptomatic very early when they are small in size (< 2 cm) and are characterized by somatic *YY1* mutations in about 30% of cases, or recurrent somatic amplifications (in particular chromosome 7 amplifications) (39).

Another subtype consists of rare clinically aggressive “atypical” insulinomas. They do express *ARX* and are characterized by large tumor size (3.5–9 cm) and metastatic behavior (40). *ARX*-positive insulinomas show genetic alterations also seen in non-functioning pNETs, such as loss of *ATRX/DAXX* and *CDKN2A*. It has been suggested that atypical insulinomas most likely exist as non-functioning pNETs for a time before becoming clinically functioning (41).

We did not perform genetic studies of tumor cells, but may suspect that one of our patients (case#12) had an “atypical” insulinoma presenting with multiple liver metastases at the time of diagnosis.

The other patient with high malignancy-potential insulinoma in our cohort (case #20; no MEN1 mutations) presented with multiple pancreatic lesions, requiring pancreatic gastroduodenal resection, but distant metastases were found eight years after the surgery. According to the literature, patients may develop metastatic disease several years after excision of insulinomas that initially were considered benign. This relapse risk is more probable in grade G2 tumors (36, 42). In our cohort, G2 differentiation grade of the tumor was found in 60% of cases. These data, together with our observation of distant metastases found almost a decade after the pancreatic gastroduodenal resection, urge for long and specific follow-up of all children with insulinoma, with or without MEN1.

Little is known on the efficacy of hyperglycemic drugs in children with insulinomas. In adults with insulinoma, diazoxide was shown to be effective in approximately 40-60% (43). According to a recent publication, children with insulinomas are less responsive to diazoxide therapy (12). In our cohort, only 10 out of 22 children received diazoxide, of which seven showed some response to it, although we do not have data on control fasting tests. Since the presence of somatostatin receptors was observed in insulinomas, treatment with somatostatin analogs has also been used in insulinoma patients (44). However, the usefulness of somatostatin analogs in the treatment of insulinoma patients remains controversial (45). In our group, somatostatin analogues were used in three patients only and did not significantly improve glycemia. Unfortunately, we lack the data on somatostatin receptors expression in these cases.

MEN1 syndrome is known to be responsible for about 4-7% of insulinoma cases in adults (1), whereas our pediatric cohort had a much higher frequency of 38%. Interestingly, all of our patents had insulinoma as the first MEN1 manifestation, while other components of the syndrome developed later in life. When comparing the main clinical and histological features of sporadic and MEN1 patients in our cohort, a tendency towards earlier onset and higher proliferative index of the lesions, as well as significantly higher number of pNETs among MEN1 cases were observed. Multiple pancreatic lesions among sporadic cases were found in one patient only (case#20), which probably represent a metastatic invasion of the pancreas rather than primary multiple tumors.

We did not observe a relapse of the insulin producing tumors during the follow-up period in sporadic or MEN1 patients. In one of the MEN1 patients (case# 5), additional pNETs were found during follow-up, leading to a second surgery at the age of 16 years where three tumors were removed. Both preoperative blood biochemical tests and postoperative immunohistochemical studies confirmed the absence of insulin secreting cells in these lesions.

Apart from the MEN1 gene, only few genes are known to cause insulinoma (14, 16). Very few reports in the literature describe the association of insulinoma with neurofibromatosis 1 and tuberous sclerosis (3–5). In our cohort, children did not present with any specific clinical features but hypoglycemia and, therefore, were tested for the MEN1 mutations only. Several studies have been performed to evaluate the possible genetic background of the tumorigenesis of insulinoma and multiple candidate genes have been identified (46). In pediatric insulinomas, aneuploidy of chromosome 11 and other chromosomes have been found to be common in both MEN1 and non-MEN1 patients (13). Further experiments are essential to validate the clinical relevance of these findings.

Limitations of this study includes its retrospective design and missing data on family history and patient’s current state. Some of the family members declined to undergo the investigations, or were not available for interview. Another limitation is the risk of type 2 statistical errors due to the extreme rareness of the disease in the pediatric population. Strengths of the study include, on the other hand, the unique high number of patients included, the detailed clinical and paraclinical work-up and the relatively long follow-up time.

## Conclusion

In this exceptionally large cohort of rare pediatric patients with primary insulinomas, we identified a high incidence of MEN1 syndrome. There was no significant difference in clinical features of sporadic and MEN1 cases, emphasizing the need for genetic testing in all children with insulinoma even in the absence of any other features. Review of the pathology results showed a high prevalence of G2 tumors in our patients. Even though malignant insulinomas are extremely rare in young children, we described the possibility of distant metastases developing many years after the diagnosis, indicating the importance of a prolonged follow up of the patients.

## Data availability statement

The raw data supporting the conclusions of this article will be made available by the authors, without undue reservation.

## Ethics statement

This study was performed in line with the principles of the Declaration of Helsinki. The studies involving human participants were reviewed and approved by the local ethics committee of The Endocrinology Research Center (protocol number 10 from 26/05/2021). Written informed consent to participate in this study was provided by the participants’ legal guardian/next of kin prior to genetic testing which was conducted as part of routine clinical care.

## Author contributions

MM, DG contributed in conception and design of the study and writing the manuscript, ASh wrote the first draft of the manuscript, AB, MK, AE, YS, SM, JA and ASt collecting data, KB and AT collecting of genetic results and analysis, HTC and KA major revision of the manuscript, LG - collecting the pathology data and analysis, major revision of the manuscript. All authors contributed to manuscript revision, read, and approved the submitted version.
